# Complete Genome Sequence of *Citrobacter freundii* Myophage Merlin

**DOI:** 10.1128/genomeA.01133-15

**Published:** 2015-10-01

**Authors:** Kayla C. LeSage, Emily C. Hargrove, Jesse L. Cahill, Eric S. Rasche, Gabriel F. Kuty Everett

**Affiliations:** Center for Phage Technology, Texas A&M University, College Station, Texas, USA

## Abstract

Bacteriophage Merlin is a T4-like myophage which infects *Citrobacter freundii*, a member of the *Enterobacteriaceae* family. *C. freundii* is an opportunistic pathogen that is a common cause of nosocomial infections. This report announces the complete genome of myophage Merlin and describes its features.

## GENOME ANNOUNCEMENT

*Citrobacter freundii* is an opportunistic pathogen that is commonly found in the environment, food, and the intestinal tracts of animals, including humans (1). *C. freundii* infections have been reported to be acquired nosocomially. The bacteria can cross the blood-brain barrier, which most commonly causes brain abscesses, neonatal meningitis, neonatal sepsis, and damage to the central nervous system, all of which have a high fatality rate (2). The persistence of *C. freundii* is primarily due to its antimicrobial resistance and imperfect hospital practices (1). To that end bacteriophages, particularly myophage Merlin, may be useful for the treatment of *C. freundii*.

Bacteriophage Merlin was isolated from a water sample collected in College Station, TX. Phage DNA was sequenced in an Illumina MiSeq 250-bp paired-end run with a 550-bp insert library at the Genomic Sequencing and Analysis Facility at the University of Texas (Austin, TX, USA). Quality controlled, trimmed reads were assembled into a single contig of circular assembly at 25.9-fold coverage using SPAdes version 3.5.0 (3). The contig was confirmed to be complete by PCR using primers that face the upstream and downstream ends of the contig. Products from the PCR amplification of the junctions of concatemeric molecules were sequenced by Sanger sequencing (Eton Bioscience, San Diego, CA). Genes were predicted using GeneMarkS (4) and corrected using software tools available on the Center for Phage Technology (CPT) Galaxy instance (https://cpt.tamu.edu/galaxy-public/). Morphology was determined using transmission electron microscopy performed at the Texas A&M University Microscopy and Imaging Center.

Merlin is T4-like phage with a 172,733-bp genome that is circularly permuted. For annotation purposes Merlin was opened to the *rIIa* gene (5). Merlin and T4 have 55.9% nucleotide sequence identity across the genome, as determined by Emboss Stretcher (6). Most of the differences between the two phages are found in hypothetical proteins of unknown function. Merlin has 303 predicted coding sequences, of which 119 (39.3%) had homologs predicted by BLASTp and InterPro Scan analysis (7, 8), 183 were conserved hypothetical proteins (60.4%), and 1 was a hypothetical novel protein. The G+C content is 38.8% with a coding density of 94.4%. Eleven tRNAs were identified in phage Merlin compared to the 8 tRNAs present in T4 (5).

T4-like core genes involved in replication, recombination, DNA packaging, lysis, and virion morphogenesis were identified with few deviations. Merlin encodes no homing endonucleases, in contrast to the 14 homing endonucleases present in T4. Merlin contains no homologs to the *ipI*, *ipII*, or *ipIII* internal head proteins of T4 and no RepEA/B replication initiation protein homologs (5). A DksA/TraR domain protein that contains a Cys4 zinc finger motif was identified. This protein is prevalent in many T4-like phages but not present in T4 itself. The primase proteins of bacteriophages T4 and T7 have a Cys4 motif that mediates protein-DNA interactions; however, the function of the Cys 4 motif in the DksA/TraR protein described here is unknown (9).

### Nucleotide sequence accession number.

The genome sequence of phage Merlin was contributed to GenBank with the accession number KT001915.
